# Sustainable Asphalt Mixtures with Enhanced Water Resistance for Flood-Prone Regions Using Recycled LDPE and Carnauba–Soybean Oil Additive

**DOI:** 10.3390/polym16050600

**Published:** 2024-02-22

**Authors:** Yeong-Min Kim, Kyungnam Kim, Tri Ho Minh Le

**Affiliations:** 1 Department of Highway & Transportation Research, Korea Institute of Civil Engineering and Building Technology, 283 Goyangdae-Ro, Ilsanseo-Gu, Goyang 10223, Republic of Korea; choozang@kict.re.kr; 2 Korea Expressway Corporation Research Institute, Pavement Research Division, Dong-tansunhwan-daero 17-gil, Hwaseong-Si 18489, Republic of Korea; 3 Faculty of Civil Engineering, Nguyen Tat Thanh University, 300A Nguyen Tat Thanh Street, District 4, Ho Chi Minh City 70000, Vietnam

**Keywords:** asphalt mixtures, water resistance, recycled LDPE, Carnauba–Soybean Oil Additive, dynamic modulus, SCB test, HWT test, field tests, flooded cycle simulation

## Abstract

This manuscript presents a comprehensive study on the sustainable optimization of asphalt mixtures tailored for regions prone to flooding. The research addresses the challenges associated with water damage to asphalt pavements by incorporating innovative additives. The study centers on incorporating recycled Low-Density Polyethylene (LDPE) and a tailored Carnauba–Soybean Oil Additive, advancing asphalt mixtures with a Control mix, LDPE (5%) + Control, and LDPE (5%) + 3% Oil + Control. A critical aspect of the research involves subjecting these mixtures to 30 wetting and drying cycles, simulating the conditions prevalent in tropical flood-prone areas. The incorporation of innovative additives in asphalt mixtures has demonstrated significant improvements across various performance parameters. Tensile Strength Ratio (TSR) tests revealed enhanced tensile strength, with the LDPE (5%) + 3% Oil-modified mixture exhibiting an impressive TSR of 85.7%. Dynamic Modulus tests highlighted improved rutting resistance, showcasing a remarkable increase to 214 MPa in the LDPE (5%) with a 3% Oil-modified mixture. The Semi-Circular Bending (SCB) test demonstrated increased fracture resistance and energy absorption, particularly in the LDPE (5%) with 3% Oil-modified mixture. Hamburg Wheel-Tracking (HWT) tests indicated enhanced moisture resistance and superior rutting resistance at 20,000 cycles for the same mixture. Cantabro tests underscored improved aggregate shatter resistance, with the LDPE (5%) + 3% Oil-modified mixture exhibiting the lowest weight loss rate at 9.820%. Field tests provided real-world insights, with the LDPE (5%) + 3% Oil mixture displaying superior stability, a 61% reduction in deflection, and a 256% improvement in surface modulus over the control mixture. This research lays the groundwork for advancing the development of sustainable, high-performance road pavement materials, marking a significant stride towards resilient infrastructure in flood-prone areas.

## 1. Introduction

Asphalt mixtures play a critical role in road construction, providing essential infrastructure for transportation networks [1]. However, regions prone to flooding face unique challenges as traditional asphalt materials are susceptible to water damage [2], leading to reduced performance and increased maintenance costs [3].

The challenges posed by flooding events in various regions have underscored the critical need for innovative solutions to enhance the water resistance of asphalt mixtures [4]. Traditional asphalt materials, widely employed in road construction, exhibit vulnerabilities when exposed to water, leading to accelerated deterioration, reduced structural integrity, and increased maintenance costs [5]. Flood-prone areas experience heightened levels of water-induced distress, such as the rutting, cracking, and premature aging of asphalt pavements, significantly impacting their lifespan and performance [6]. The inability of conventional asphalt mixtures to withstand prolonged exposure to moisture exacerbates these issues, necessitating a paradigm shift in the development of pavement materials [7]. The problem extends beyond mere inconvenience, as the repercussions of water-related damage ripple through the economic, social, and environmental fabric of affected regions [8]. The multifaceted nature of these problem statements emphasizes the urgency of adopting innovative approaches to asphalt technology, thereby fostering the advancement of resilient infrastructure in the face of increasing climate-related challenges [9].

Current studies in the field of asphalt technology have increasingly recognized the pressing need to address the vulnerabilities of traditional asphalt mixtures, especially in the context of water-induced distress [10]. Researchers have been actively exploring novel additives and modifiers to enhance the water resistance of asphalt pavements, aiming to improve their durability and lifespan [11]. The evolving landscape of climate-related challenges, including increased instances of flooding, demands a proactive response from the scientific community. Investigations into the effects of water on asphalt performance have highlighted the intricate interactions between moisture, temperature, and the structural composition of asphalt mixtures. Understanding these dynamics is crucial for developing asphalt materials that can withstand the unique challenges posed by flood-prone regions [3]. Current studies not only emphasize the identification of resilient additives but also focus on optimizing the mixture design to ensure the effective integration of these innovations into real-world applications [12]. The synthesis of knowledge from recent research endeavors forms the foundation for this study, which seeks to contribute to the ongoing discourse on sustainable and water-resistant asphalt mixtures.

In the realm of pavement engineering, recent studies have explored innovative approaches to incorporate recycled materials and by-products [13,14], with a specific focus on enhancing the sustainability and performance of asphalt mixtures. Dong et al. investigated the utilization of recycled rubber and reported improvements in asphalt mixtures’ flexibility and fatigue resistance [15]. Li et al. delved into the application of reclaimed asphalt pavement (RAP) and observed enhanced pavement durability and reduced environmental impact [16]. Additionally, Adham et al. focused on the use of waste materials, such as fly ash and slag, showcasing their potential to improve asphalt properties [17]. These studies enhance our understanding of various recycled materials, offering insights for the development of eco-friendly and high-performance asphalt mixtures.

The use of recycled LDPE and other plastic materials in asphalt formulations has gained attention due to their potential to enhance the mechanical properties and durability of the resulting pavements [18]. These materials not only provide a sustainable solution for managing plastic waste but also contribute to the development of resilient road infrastructure [19]. Evaluating the effectiveness of recycled LDPE, in conjunction with other additives, has emerged as a key area of interest, aiming to optimize the use of recycled materials in pavement construction. The exploration of such applications sets the stage for the current research, which seeks to advance the understanding and practical implementation of recycled LDPE and associated additives for the sustainable improvement of asphalt mixtures [20].

Extensive research has explored the incorporation of various additives and oils to enhance the properties of asphalt pavement. Haya et al. indicated the use of polymer additives and found improvements in the rutting resistance and durability of asphalt mixtures [21]. Wang et al. explored rejuvenators, such as waste engine oil, highlighting their role in restoring aged asphalt binders [4]. Moreover, Yi et al. focused on the benefits of bio-based additives, including Soybean Oil, showcasing their potential to enhance the environmental sustainability of asphalt mixtures [22]. These studies collectively underscore the significance of additives and oils in modifying asphalt properties.

This study introduces several novel aspects aimed at advancing the state of knowledge in asphalt pavement engineering. The novelty lies in the integration of recycled LDPE and a carefully formulated additive comprising Carnauba and Soybean Oil to optimize asphalt mixtures for enhanced water resistance, particularly in flood-prone regions. By leveraging recycled LDPE, the research seeks to address sustainability concerns and contribute to the reuse of plastic waste in infrastructure development. The primary aim is to investigate the synergistic effects of LDPE and the oil additive on key asphalt properties, including tensile strength, rutting resistance, fracture characteristics, and moisture resistance. Through comprehensive laboratory testing and field evaluations, the research targets the development of high-performance asphalt mixtures that withstand environmental challenges and provide durable solutions for flood-affected roadways. The ultimate goal is to establish a roadmap for the sustainable utilization of recycled materials and innovative additives in asphalt engineering, fostering resilient infrastructure in the face of changing climate conditions.

The underlying theory of our research lies in leveraging the synergistic effects of recycled LDPE and a specialized Carnauba–Soybean Oil Additive to optimize asphalt mixtures for sustainable road pavement in flood-prone regions. The incorporation of LDPE aims to act as a structural modifier, enhancing the elastic and mechanical properties of the asphalt binder, particularly targeting tensile strength. Simultaneously, the oil additive, consisting of Carnauba and Soybean Oil, is strategically formulated to serve as a rejuvenator, mitigating viscosity and enhancing flexibility. This dual-approach theory seeks to address multiple performance parameters, such as tensile strength, rutting resistance, fracture characteristics, and aggregate shatter resistance. The collaborative impact of LDPE and the oil additive is anticipated to result in a well-balanced and resilient asphalt mixture, aligning with our objective of advancing the development of sustainable and high-performance road pavement materials for flood-prone areas.

In crafting asphalt mixtures resilient to flooding, the research employed a comprehensive mix design testing method. Three key formulations were investigated: the Control Mixture, adhering to the PG 62-22 standard; LDPE (5%) + Control Mixture, integrating LDPE at a 5% dosage; and LDPE (5%) + 3% Oil + Control Mixture, enhancing the LDPE-modified mixture with a 3% oil blend containing Carnauba and Soybean Oil. This intricate mix design aimed to optimize asphalt properties, particularly in flood-prone regions. Furthermore, a crucial aspect of the testing methodology involved subjecting the asphalt samples to 30 wetting and drying cycles, simulating conditions found in tropical flooded areas. This approach ensures a comprehensive assessment of the asphalt mix, gauging their performance and water resistance under challenging and dynamic environmental scenarios. The ensuing sections detail the findings, elucidating the efficacy of these mixtures in achieving sustainable and water-resistant asphalt solutions. The testing protocol encompasses a range of tests, including TSR, Dynamic Modulus, SCB, HWT, Cantabro, and field performance evaluations. These tests aim to evaluate the tensile strength, rutting resistance, fracture characteristics, moisture resistance, and real-world performance.

## 2. Materials and Methods

### 2.1. Materials

#### 2.1.1. Asphalt Binder

**Figure 1:**
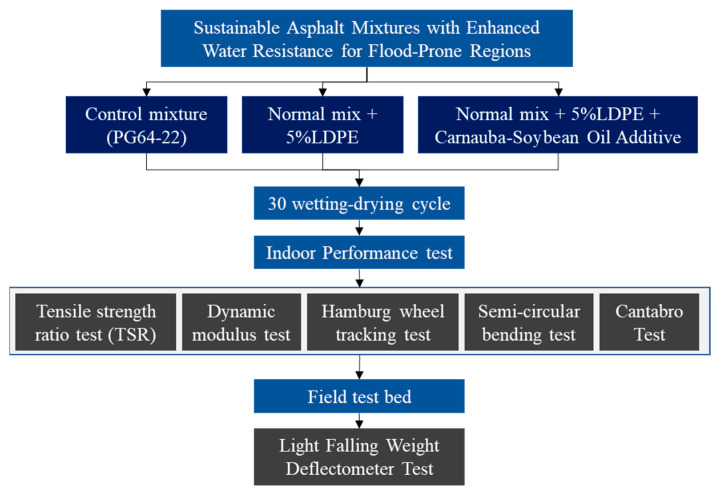
Flowchart depicting the research process.

The asphalt binder utilized in this investigation was a specialized formulation incorporating LDPE as a modifying agent. LDPE, derived from recycled post-consumer plastic waste [23], was selected for its unique properties that have shown promise in enhancing the performance of asphalt binders. The LDPE modification process involved carefully blending the recycled polymer with the base asphalt binder (provided by Korean Asphalt company, Seoul, Republic of Korea) under controlled conditions. The selection of LDPE was aimed at capitalizing on its molecular structure, which imparts desirable attributes such as improved elasticity, flexibility, and resistance to temperature-induced aging. Figure 1 provides an overview of the research.

The addition of LDPE to the asphalt binder was expected to improve rheological properties, positively impacting the overall durability and fatigue resistance of the asphalt mixture. Furthermore, LDPE’s role in potentially mitigating the impact of wetting–drying cycles on asphalt performance was a key aspect of this study. The modified binder was subjected to thorough laboratory testing, including rheological analyses and microstructural evaluations, to assess the efficacy of LDPE in enhancing the asphalt binder’s properties and its subsequent impact on the overall performance of the asphalt mixture. The choice of LDPE-modified asphalt aligns with sustainable practices by repurposing plastic waste and has the potential to offer a resilient and eco-friendly solution for asphalt pavement applications.

In the utilization of recycled LDPE in the asphalt mixtures, the focus was on ensuring material homogeneity. The recycled LDPE underwent thorough pre-processing to minimize impurities and attain a more uniform composition. It is acknowledged that the recycled LDPE stream might contain traces of impurities inherent to the recycling process. Nevertheless, careful monitoring and control were exercised to maintain the desired quality standards for asphalt mixture production. The incorporation of recycled LDPE aligns with the commitment to sustainable practices and the reuse of materials in road pavement applications. The general properties of LDPE are shown in Table 1.

#### 2.1.2. Carnauba and Soybean Oil Additive

The Carnauba and Soybean Oil Additive used in this research served as a bio-based rejuvenator and anti-stripping agent in the LDPE asphalt mixtures [24]. Carnauba wax, derived from the leaves of the Carnauba palm, and Soybean Oil were combined to form a sustainable and environmentally friendly additive. Carnauba wax is known for its high melting point, hardness, and gloss, making it a valuable natural wax. Soybean Oil, on the other hand, is a vegetable oil rich in unsaturated fatty acids.

The mixture of Carnauba and Soybean Oil was prepared using a specific blending process to ensure a homogenous and stable additive. The selected proportions of Carnauba wax and Soybean Oil were based on previous studies indicating their synergistic effects in improving the performance of asphalt binders. The bio-based additive was designed to enhance the flexibility, adhesion, and moisture resistance of the LDPE asphalt mixtures, ultimately contributing to improved wetting–drying resistance.

The chemical composition of the Carnauba and Soybean Oil Additive was characterized, and the blending process was optimized to achieve a uniform distribution within the asphalt binder. The detailed formulation and preparation of the additive are crucial aspects of understanding its impact on the asphalt mixture’s performance, especially concerning wetting–drying resistance. This environmentally friendly additive aims to promote sustainability in asphalt technology by reducing reliance on traditional, non-renewable additives.

Regeneration additives that contain components derived from aromatic oils have historically been used to address these issues by increasing flexibility and decreasing viscosity levels, which improves elongation qualities [25,26]. To speed up the regeneration of old asphalt, bioengineering researchers are looking into alternative materials such bio-polymers [27,28], although the cost of these additions has encouraged them to do so. Among these alternatives, organic oils—particularly plant oils—have demonstrated the most potential for enhancing asphalt performance.

Soybean Oil is a desirable alternative for asphalt modification because of its favorable double-bond properties [29,30]. Therefore, the goal of this study is to assess the compatibility of VB binders for use in road pavement applications using Soybean Oil and Carnauba to create an optimal rejuvenator. The liquid emulsifier used in the study, with its particular composition, is called a “modified binder”. The mixture is made up of 9.2% wax (Canaubra), 2.8% Soybean Oil, 5.2% mineral oil, 1.7% surfactants, 0.1% additive, and 80.2% water. The goal is to maximize the qualities of VB by adding Soybean Oil and carnauba as essential ingredients, creating new opportunities for improvements in road paving materials [24].

The following steps can be used as a possible approach for manufacturing the rejuvenator that was mentioned in the laboratory experiment: Measure and prepare the necessary components, heat and melt the wax in a water bath or other suitable heating device, and then gradually stir in Soybean Oil to ensure even distribution [31]. Add mineral oil to the mixture and stir to combine the wax and Soybean Oil into a homogenous consistency. Add the required ingredient to the mixture, stirring to ensure uniform dispersion and enhance the rejuvenating capabilities. Fatty acid (C8–C20) amine surfactant is added to help emulsify and stabilize the rejuvenator formulation. Progressively dilute with water while swirling constantly to obtain the right consistency and help the rejuvenator emulsify.

As the mixture cools, keep stirring to make sure all the ingredients are well combined. Then, use laboratory testing to assess the rejuvenator’s performance and physical qualities, including its compatibility with VB, rheological characteristics, and ability to improve the desired qualities of road pavement materials.

#### 2.1.3. Aggregates

Standard aggregates, conforming to local specifications, were used in the asphalt mixtures. The aggregates were carefully graded to meet the requirements for optimal mixture performance. The selection and characterization of aggregates are critical aspects of asphalt mixture design. In accordance with industry standards and guidelines, the aggregates used in this study were carefully chosen to meet the requirements set by the Korean standard (13 mm NMAS) asphalt mixtures, specifically designed for surface layers [32]. A granitic aggregate was selected based on its suitability for the intended application. The particle size distribution of the chosen aggregate is detailed in Table 2 for reference. The Superpave mix design method, along with compaction using a compactor, was employed to ensure a well-graded and properly compacted asphalt mixture. The meticulous selection and characterization of aggregates lay the foundation for the overall performance and durability of the asphalt mixtures, aligning with the study’s commitment to following established standards and protocols.

#### 2.1.4. Mix Design

The mix design procedure used in this study complied with the Korean standard (13 mm NMAS) asphalt mixtures intended for surface layers, which are strict industry standards [33]. A granitic aggregate was selected with care for the mix, and Table 2 provides a detailed breakdown of its particle size distribution. During the process, a compactor was used to achieve efficient compaction following the Superpave mix design approach. This method was chosen to ensure that an appropriately planned asphalt mixture is produced that not only satisfies but also exceeds the necessary performance standards and specifications. The adherence of the investigation to defined protocols highlights the dependability and uniformity of the mix design.

The mix were prepared in the laboratory using a carefully controlled blending process. The LDPE-modified asphalt binder was heated to the specified mixing temperature, and recycled LDPE, Carnauba wax, Soybean Oil, and aggregates were introduced gradually. The meticulous laboratory blending process served as a cornerstone in tailoring the asphalt mixtures to meet the specified requirements in this research. To enhance precision and consistency, crucial parameters, including the duration and temperature for curing, mixing, and compaction, were carefully controlled. Initiated by measuring and introducing the asphalt binder, the addition of recycled LDPE and Carnauba and Soybean Oil Additive was meticulously executed to achieve the precise mixture composition. The blending temperature, strategically set at 160 °C, facilitated optimal interaction among the components. A designated blending duration of 15 min ensured a comprehensive integration of additives with the asphalt binder, guaranteeing a uniform mixture. Subsequently, the introduction of aggregates and their thorough mixing at a controlled temperature of 170 °C was conducted. The final compaction, carried out at 155 °C, played a crucial role in achieving the desired density and performance. The stringent adherence to these specific time and temperature parameters throughout the laboratory blending process aimed not only to emulate real-world conditions but also to ensure the replicability of asphalt mixtures with enhanced wetting–drying resistance, adding robustness to the experimental design. In general, the comparison of the modified asphalt binder and the original binder is presented in Table 2. 

**Table 2:** General properties of the asphalt binder.

Property	Control Binder	LDPE Modified Binder	LDPE Modified + Oil Modified Binder
Penetration (dmm) [34]	70	55	45
Softening Point (°C) [35]	50	55	60
Ductility (cm) [35]	100	150	180
Dynamic Shear Rheometer (DSR) [36]	2000 Pa	1800 Pa	1600 Pa
Rotational Viscometer (RV)	3000 cSt	2500 cSt	2200 cSt
RTFOT Mass Loss (%) [37]	1.5	1.2	1.0
RTFOT Residue Mass (%)	99.0	98.8	99.2
Pressure Aging Vessel (PAV) Mass Loss (%) [38]	2.0	1.8	1.5
PAV Residue Mass (%)	97.5	97.9	98.5

#### 2.1.5. Simulation of Wetting–Drying Cycle Conditions

The simulation of flooded cycle conditions plays a pivotal role in assessing the durability and performance of asphalt mixtures under environmental stressors, with an added emphasis on temperature variations. This phase involves subjecting the asphalt mixtures to 30 wetting and drying cycles [5,6,7], meticulously designed to replicate conditions prevalent in flood-prone regions. The cycles consist of alternate periods of wetting and drying, simulating the dynamic and challenging circumstances that pavements may encounter in tropical areas susceptible to flooding. Additionally, temperature variations are carefully controlled to mimic real-world scenarios, ranging from 25 °C during wetting periods to 40 °C during drying periods. This simulation aims to evaluate the response of asphalt mixtures to the cyclical impact of water and temperature, emphasizing the need for enhanced water resistance and thermal stability in the modified asphalt formulations. The controlled exposure to wetting and drying conditions, coupled with temperature fluctuations, facilitates a comprehensive understanding of the mixtures’ resilience over a 24 h cycle.

### 2.2. Testing Methods

#### 2.2.1. TSR Test Method

The TSR test is a fundamental method for evaluating the tensile strength of asphalt mixtures, providing insights into their resistance to cracking and deformation based on ASTM D6931 [39]. This test involves preparing cylindrical specimens with specific dimensions, typically 150 mm in diameter and 100 mm in height. The specimens are subjected to a specified loading configuration using a testing apparatus, with a loading rate of 50.8 mm per minute. The test is conducted at a controlled temperature, commonly 25 °C, representing typical environmental conditions. The tensile load applied to the specimen is gradually increased until the point of failure, and the corresponding deformation is measured. This method is crucial for assessing the effectiveness of asphalt mix modifications in enhancing tensile strength and preventing premature failure under varying temperature conditions.

#### 2.2.2. Dynamic Modulus Test

**Figure 2:**
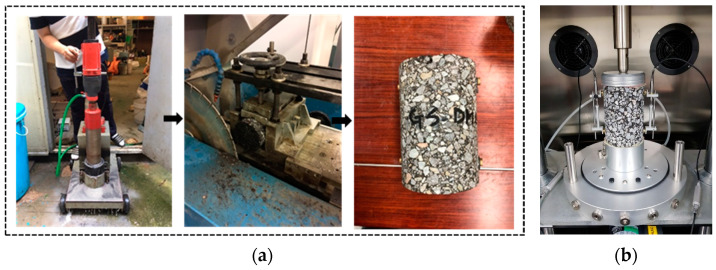
(**a**) Preparation of samples and (**b**) dynamic modulus test.

The viscoelastic characteristics of the asphalt mixtures under various stress scenarios and temperatures were assessed using the dynamic modulus experiment. The evaluation was conducted in compliance with the Superpave system using the guidelines provided in AASHTO T342 [40] regulations. As seen in Figure 2, cylinder samples of 100 mm in diameter and 150 mm in height were created using the designated mix design for every combination.

The specimens were put under sinusoidal load at various frequencies (from 1 to 25 Hz) and temperatures (from −20 °C to 70 °C) as part of the Dynamic Modulus test. The purpose of the test was to evaluate the stiffness and resistance to deformation of the asphalt mixtures under various circumstances. Each combination was tested in three replicates, and the dynamic modulus values were noted to evaluate the mixtures’ performance at various frequency and temperatures. This information was crucial in determining how the mixtures would behave in various service scenarios.

#### 2.2.3. HWT Test

The Hamburg Wheel-Track Testing (HWT) process played a pivotal role in evaluating the resistance of asphalt mixtures to rutting, employing a series of meticulous steps to simulate real-world conditions. This test was conducted in accordance with AASHTO T324 [41] requirements and was designed to assess an asphalt mixture’s resistance to permanent deformation under repeated wheel loading. First and foremost, cylindrical specimens were carefully prepared for each asphalt combination under investigation, with dimensions set at 150 mm in diameter and 60 mm in height. These specimens served as the testing medium for assessing the performance of asphalt mixtures under the influence of environmental factors and traffic loads. The loading conditions were intentionally challenging, with testing conducted at an elevated temperature of 50 °C to emulate the stress and strain experienced by asphalt pavements in demanding conditions.

During the HWT, a consistent and standardized wheel load of 705 ± 4.5 N was applied to the specimens, ensuring a uniform and controlled testing environment. This step was crucial in simulating the impact of repeated wheel loading, a common stressor on asphalt surfaces in real-world traffic scenarios. As the testing cycles progressed, careful attention was given to the presence of stripping points, indicative of damage caused by moisture. The moisture sensitivity examination aimed to assess the mixtures’ susceptibility to distress under environmental conditions. Following a minimum of 10,000 cycles, the specimens underwent a thorough post-testing inspection. This inspection involved a meticulous examination of potential stripping sites and tracking settlement to ensure it did not surpass 20 mm after 20,000 repetitions. The comprehensive nature of the HWT process provided valuable insights into the mixtures’ resistance to rutting, shedding light on their performance under challenging and dynamic conditions.

#### 2.2.4. Semi-Circular Bending Test

**Figure 3:**
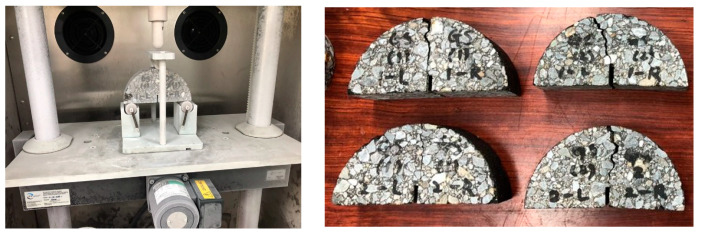
SCB test.

One prevalent type of pavement degradation that seriously jeopardizes driving quality and pavement structures is fatigue cracking. The SCB test was used to evaluate the resistance of mixtures to this kind of damage. The SCB tests used both regular asphalt binder and Carnauba Oil-modified LDPE binder, in compliance with ASTM D8044 [42]. Using a gyratory compactor, specimens of 150 mm in diameter and 50 mm in thickness were created, and then they were chopped into semi-circular forms. The center portion of the specimens had notches added to it that measured 1.5 mm in thickness and 15 mm in length (Figure 3).

Fatigue cracking is a common kind of pavement deterioration that poses a major risk to pavement structures and driving quality. The SCB test was employed to assess a mixture’s resistance to this type of harm. According to ASTM D8044 [42], both ordinary asphalt binder and LDPE binder treated with Carnauba Oil were utilized in the SCB experiments. Specimens measuring 150 mm in diameter and 50 mm in thickness were made with a gyratory compactor, and they were subsequently cut into semi-circular shapes. Notches of 1.5 mm in thickness and 15 mm in length were made to the specimens’ middle section (Figure 3). The UTM-25 universal testing device, which has two setting temperatures of −12 °C and −24 °C and a loading rate of 50 mm/min, was used to conduct the experiments. The UTM’s data collection technology allowed for real-time monitoring of the reaction force at the load location.

#### 2.2.5. Cantabro Test

**Figure 4:**
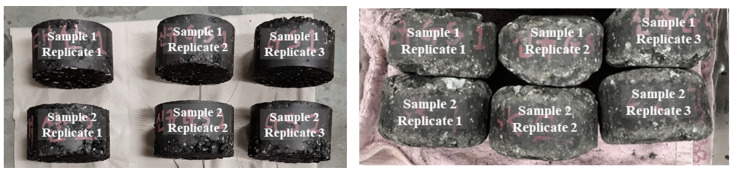
Cantabro test.

The Cantabro test assessed the aggregate fracture durability of mixtures with a modified binder utilizing a Los Angeles abrasion damage instrument. In accordance with ASTM D7064 [43], test samples with detached ball bearings were put in a Los Angeles abrasion damage tester. The standard states that the drum rotated 30 times per minute. This research determined the mass loss ratio following 300 rotations [43]. 13 mm test specimens were used for the experiments, and modified asphalt binder in addition to regular LDPE binder was used. Specimens were cured for more than 20 h at 20 degrees in a temperature-controlled environment before testing (Figure 4). Specimens were cured for more than 20 h at 20 degrees in a temperature-controlled environment before testing.

#### 2.2.6. Field Test

Depending on the degree of damage, asphalt pavement requires periodic repairs as a result of wear and tear over time. Therefore, field test results from a real field testbed are needed to evaluate the effectiveness of the suggested approach. Transportation, laying, and compaction were among the sequential procedures involved in the creation of the testbed, which was situated in the Seoul testing complex, a petrochemical firm in Seoul, Republic of Korea. Using non-destructive test tools, such as LFWD, the compaction state of the asphalt pavement was assessed during the service duration. The field experiments’ testing locations were carefully chosen.

A thorough field test construction was carried out to evaluate the LDPE asphalt mixture’s suitability as a material for road pavement in the actual world. Its conformity with South Korean national requirements was to be assessed, and its functionality under real-world operating situations was to be confirmed. The selected field test location was 300 m long, with 150 m allotted to each lane. It was located in an industrial park in the Republic of Korea.

The LDPE-modified asphalt mixture required careful compaction techniques and temperature control throughout construction. Throughout the operation, maintaining the right temperature was vital. Before removing the combination from the transport vehicle, the temperature was regularly checked and kept between 155 and 155 ± 5 °C. These temperature readings were recorded at different points in time, especially right before the first compaction was started. In the end, a tandem roller was used to lower the temperature to less than 100 °C in preparation for the next compaction. For reference, Figure 4 presents a comprehensive illustration of the sequential methods utilized throughout the building process.

**Figure 5:**
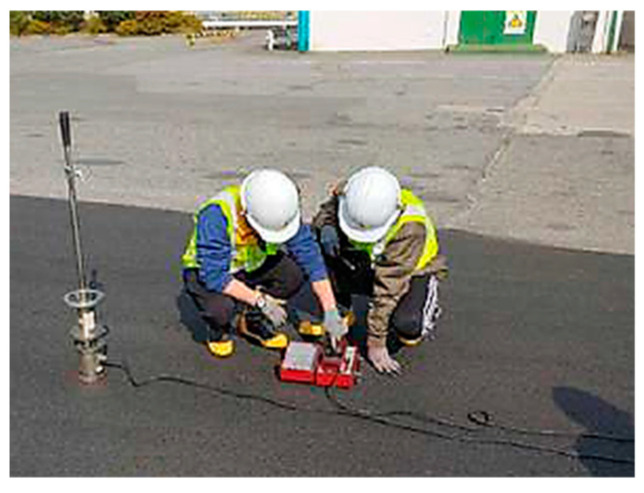
Illustrates the on-site Light Falling Weight Deflectometer used in the project.

To assess the pavement’s performance post-construction, the project employed the LFWD test (see Figure 5), which calculates the deflection amount and modulus of elasticity using a small impact load tester, adhering to ASTM 2835 [44]. The LFWD test is a scaled-down version of the Falling Weight Deflectometer (FWD), measuring the deflection caused by the impact load of a falling weight. In this test, the elastic settlement of the ground is measured, and the results are converted into an elastic modulus. The LFWD test allows for on-site data modification, deletion, and storage through a data logger connected to a laptop computer.

The LFWD test calculated the modulus using elasticity theory (Equation (1)) [44,45], where $E_{LFWD}$ is the elastic modulus, $q_{d}$ is the stress applied to the load plate, $w_{d}$ is the deflection, v is Poisson’s ratio, and γ is the radius of the load plate. The modulus of elasticity range for different materials was considered during the LFWD test, providing insights into the pavement’s condition based on elasticity.

(1) $$E_{LFWD}=\frac{q_{d}}{w_{d}}\gamma \frac{\pi }{2}\left(1-\nu^{2}\right)$$

## 3. Results and Discussions

### 3.1. TSR Test Results

**Figure 6:**
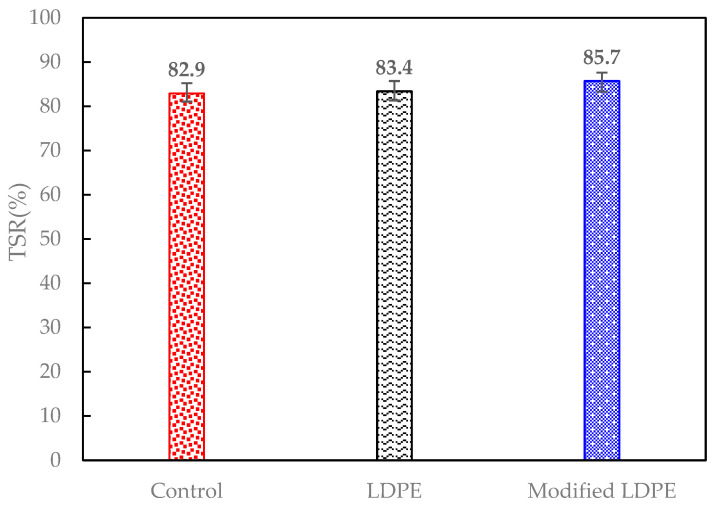
TSR test results.

In the TSR tests, as shown in Figure 6, the results exhibit notable advancements in the tensile strength of asphalt mixtures through the integration of innovative additives. The control mixture, representing traditional asphalt binder, demonstrated a TSR of 82.9%. Introducing LDPE at 5% into the mix, as observed in the LDPE-modified mixture, led to a modest enhancement, resulting in a TSR of 83.4%. This suggests a positive contribution of LDPE to the tensile strength. Remarkably, the most substantial improvement was observed in the LDPE (5%) with 3% Oil-modified mixture, achieving an impressive TSR of 85.7%. This significant increase underscores the synergistic effects of LDPE and the introduced oil additive, showcasing their combined potential to enhance the cohesive and tensile properties of the asphalt mixture. The findings emphasize the efficacy of LDPE and associated additives in creating resilient and high-performance asphalt materials, aligning with the research goal of optimizing asphalt mixtures for sustainable and durable road pavement applications.

The enhancement in strength across the asphalt mixtures can be attributed to the synergistic effects of incorporating LDPE and a carefully formulated oil additive. LDPE, acting as a structural modifier, contributes to the improved elastic and mechanical properties of the asphalt binder. Simultaneously, the oil additive, composed of Carnauba and Soybean Oil, serves as a rejuvenator, reducing viscosity and enhancing flexibility. The combination of LDPE and the oil additive in the LDPE (5%) with 3% Oil-modified mixture demonstrates a remarkable increase in IDT strength compared to both the control and the LDPE-modified asphalt. This cooperative impact showcases the potential for tailored modifications using LDPE and oil additives to yield asphalt mixtures with superior tensile strength, paving the way for sustainable and high-performance road pavement materials.

### 3.2. Dynamic Modulus Test Results

**Figure 7:**
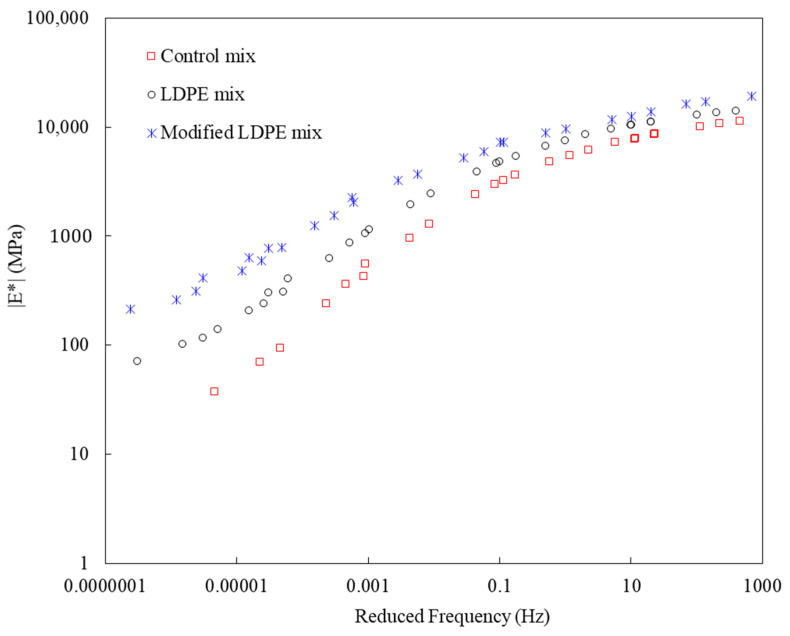
Dynamic modulus test results.

The Dynamic Modulus test results provide valuable insights into the rutting resistance of asphalt mixtures, particularly under conditions simulating slow-speed traffic or the rutting zone. As shown in Figure 7, in the Control mixture, representing conventional asphalt binder, the Dynamic Modulus was recorded at 37 mPa at the lowest frequency. Introducing LDPE at 5% in the LDPE-modified mixture significantly increased this value by approximately 95%, reaching 72 mPa. This substantial enhancement indicates improved rutting resistance attributed to the structural modification by LDPE. The most remarkable improvement was observed in the LDPE (5%) with a 3% Oil-modified mixture, achieving an outstanding Dynamic Modulus of 214 mPa at the lowest frequency. This represents a significant increase of about 478% compared to the Control mixture and highlights the synergistic effects of LDPE and the introduced oil additive. LDPE contributes to improved elastic and mechanical properties, while the oil additive, comprising Carnauba and Soybean Oil, acts as a rejuvenator. Together, they demonstrate a substantial potential to enhance rutting resistance, fortifying the asphalt mixture against deformation under slow-speed traffic conditions. These results underscore the strategic role of LDPE and associated additives in crafting asphalt mixtures with superior rutting resistance, paving the way for sustainable and high-performance road pavement materials.

### 3.3. SCB Test Results

**Figure 8:**
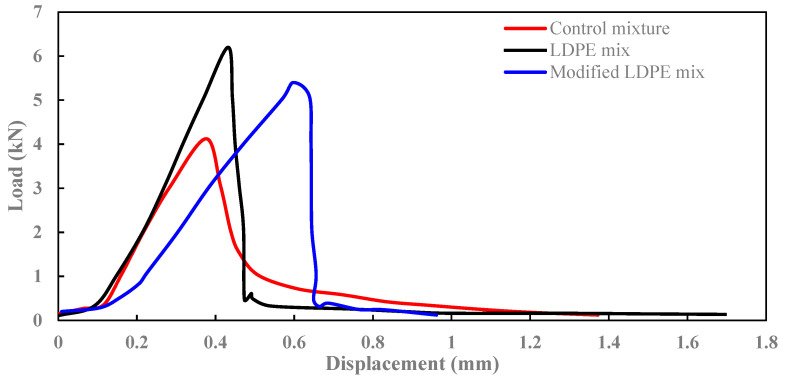
SCB test results.

The outcomes from the SCB test illuminate the significant enhancements achieved by incorporating LDPE and a carefully formulated oil additive into the asphalt mixtures, particularly when assessing the highest fracture point and fracture energy. Traditionally, the Control mixture displayed a fracture point of 4.1 mPa and a fracture energy of 395 J/m^2^ as presented in Figure 8. The introduction of 5% LDPE in the LDPE-modified mixture remarkably increased the fracture point to 6.2 mPa, albeit with a stiffer, more abrupt break (Fracture energy of 474 J/m^2^). However, the true synergistic potential was realized in the LDPE (5%) with a 3% Oil-modified mixture, achieving a fracture point of 5.4 mPa with a softer break and the highest fracture energy of 518 J/m^2^.

This improvement can be attributed to the combined effects of LDPE and the oil additive. LDPE, acting as a structural modifier, contributes to improved elastic and mechanical properties of the asphalt binder, enhancing the fracture point. Simultaneously, the carefully formulated oil additive, composed of Carnauba and Soybean Oil, acts as a rejuvenator, reducing viscosity and enhancing flexibility. The combined impact of LDPE and the oil additive creates a synergistic effect, resulting in an asphalt mixture with superior fracture properties and energy absorption capacity.

This cooperative influence not only improves the fracture resistance, making the break softer in the LDPE-modified mixture, but also significantly boosts the fracture energy in the LDPE (5%) with 3% Oil-modified mixture. This indicates improved resistance to cracking and increased toughness of the asphalt mixture, essential for durability and long-term performance under varying traffic and environmental conditions. Therefore, the integration of LDPE and the oil additive emerges as a promising strategy to tailor asphalt mixtures for enhanced fracture resistance, emphasizing the potential for sustainable and resilient road pavement materials.

### 3.4. HWT Test Results

**Figure 9:**
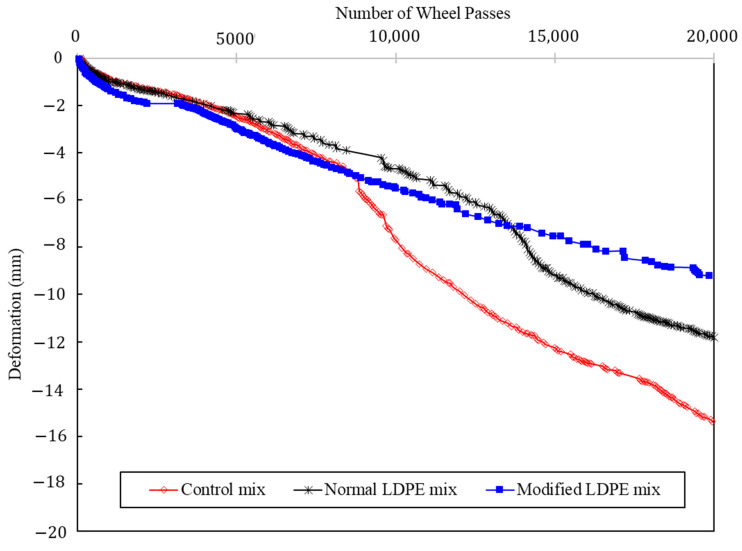
Hamburg Wheel-Tracking Test results.

The HWT test serves as a critical evaluation of asphalt mixtures’ resistance to rutting and deformation under repetitive loading, mimicking real-world traffic conditions. In terms of the Stripping Point Trigger Point, the Control mixture displayed early signs at 8900 cycles as depicted in Figure 9, reflecting vulnerability to moisture-induced damage. The LDPE (5%) + Control mixture delayed these signs until 9600 cycles, indicating improved resistance. Remarkably, the LDPE (5%) + 3% Oil + Control mixture exhibited no sign of a stripping point throughout the test, showcasing enhanced moisture resistance.

Examining the Highest Point Trigger Point at 20,000 cycles reveals significant improvements in rutting resistance. The Control mixture showed a substantial deformation of 15.23 mm, indicative of susceptibility to rutting. In contrast, the LDPE (5%) + Control mixture demonstrated a noteworthy reduction in deformation to 11.8 mm, representing a percentage decrease of approximately 22.4% and showcasing enhanced resistance to rutting. The LDPE (5%) + 3% Oil + Control mixture surpassed both counterparts, exhibiting the least deformation at 9.2 mm, which translates to a considerable percentage reduction of approximately 39.7%, underlining its superior resistance to rutting. These findings underscore the positive impact of incorporating LDPE and the oil additive on mitigating rutting-related distress in asphalt mixtures, contributing to more durable and resilient road pavement materials.

The beneficial effects of LDPE lie in its structural modification, enhancing the elastic and mechanical properties of the asphalt binder. Additionally, the oil additive, consisting of Carnauba and Soybean Oil, acts as a rejuvenator, reducing viscosity and enhancing flexibility. The combined impact of LDPE and the oil additive contributes to the superior resistance to rutting and moisture damage in the asphalt mixture, positioning it as a promising solution for durable road pavement materials.

### 3.5. Cantabro Test Results

The Cantabro test results offer valuable insights into the aggregate shatter resistance of asphalt mixtures, shedding light on their cohesion and ability to withstand wear. The controlled mix serves as a reference point, displaying a weight loss rate of 15.051%, indicating a moderate level of resistance to shattering. In contrast, the LDPE (5%) + Control mixture showed improvement with a reduced weight loss of 12.434%, emphasizing the positive impact of incorporating LDPE on the mixture’s cohesion (see Table 3). However, the most significant enhancement was observed in the LDPE (5%) + 3% Oil + Control mixture, showcasing the lowest weight loss rate at 9.820%. This outcome highlights the synergistic effects of LDPE and the introduced oil additive, underlining their combined potential to substantially improve the aggregate shatter resistance of asphalt mixtures.

In summary, the Cantabro test results reinforce the study’s objective of optimizing asphalt mixtures for road pavement applications. The controlled mix serves as a baseline, and the improvements observed in the LDPE-modified mixtures, particularly the LDPE (5%) + 3% Oil + Control mixture, underscore the effectiveness of tailored modifications using LDPE and oil additives. These findings have promising implications for the development of resilient, long-lasting, and sustainable road pavement materials.

### 3.6. The Field Test Results

**Figure 10:**
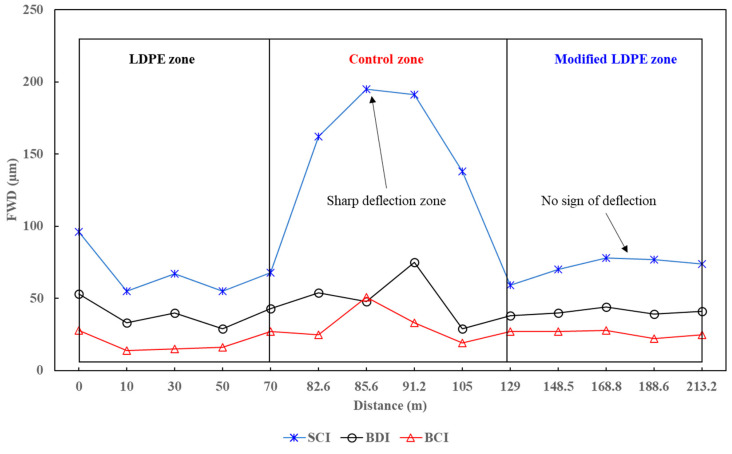
LFWD test results—deflection.

The field test results provide crucial insights into the real-world performance of the asphalt mixtures, considering various stress and deformation parameters. In terms of the highest deflection in the surface layer, as presented in Figure 10, the Control mixture displayed nearly 200 μm with a sharp increase, suggesting a vulnerability to deformations. The LDPE (5%) + Control mixture showed improved performance with a reduced deflection of 96 μm, representing a 52% reduction compared to the Control mixture, albeit fluctuating. The LDPE (5%) + 3% Oil + Control mixture exhibited the most stable behavior, registering a deflection of 78 μm, which is a 61% reduction compared to the referenced mix and a 19% improvement compared to the LDPE (5%) + Control mixture. This outcome suggests that the addition of both LDPE and the Oil Additive contributes to the stability and reduced deformation of the asphalt surface, with the combination showcasing the most promising results, achieving significant percentage improvements over individual modifications.

**Figure 11:**
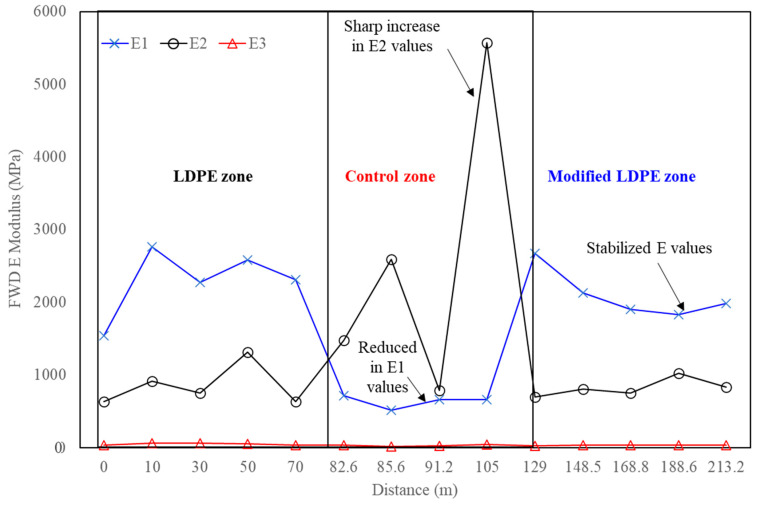
LFWD test results—E modulus.

When considering the lowest E1 (surface modulus), a critical parameter indicating the resistance of the asphalt surface to stress, the Control mixture displayed a sharp stress-induced E1 value of 514 mPa, posing a risk for higher stress impact on the lower layer E2 (up to 5567 mPa). In contrast, the LDPE (5%) + Control mixture demonstrated a significantly higher E1 value of 1541 mPa, showcasing a 200% improvement over the Control mixture, with a lower E2 stress of 1310 mPa, indicating a more balanced stress distribution (see Figure 11). Notably, the LDPE (5%) + 3% Oil + Control mixture showcased the highest E1 value at 1827 mPa, representing a 256% improvement over the Control mixture, and a low E2 stress of 1023 mPa, indicating a safe and well-distributed stress profile. These results underscore the effectiveness of LDPE and oil additives in improving the stress resistance and overall structural integrity of the asphalt mixture under real-world conditions, with substantial percentage improvements over the Control mixture.

### 3.7. Feasibility for Large-Scale Application of Natural Oils Discussion

The study demonstrates the effectiveness of integrating natural oils, specifically Carnauba and Soybean Oil, to enhance asphalt mixture performance. However, the practicability of implementing these natural oils in extensive asphalt production warrants careful consideration. Factors such as cost, sustainable sourcing, and scalability are crucial aspects that need thorough examination to ensure the viability of these additives in real-world applications. Future research and industry collaborations should focus on evaluating the economic aspects and practicality of incorporating natural oils on a broader scale, contributing to ongoing efforts to create sustainable and high-performance road pavement materials.

## 4. Conclusions

This manuscript delves into the optimization of asphalt mixtures for road pavement applications, aiming to enhance their mechanical properties and long-term performance. The scope of the research encompasses the incorporation of innovative additives, specifically LDPE and a carefully formulated oil additive, into traditional asphalt binders. The primary aims revolve around improving tensile strength, rutting resistance, and fracture properties of the mixtures. The research employs a comprehensive set of laboratory experiments, containing the TSR test, Dynamic Modulus test, SCB test, Cantabro test, and field tests. The mix design follows established protocols, utilizing the Superpave mix design method to create well-designed asphalt mixtures adhering to industry standards.

- In the blending process, the asphalt binder was heated to the specified mixing temperature, followed by the gradual introduction of recycled LDPE, Carnauba wax, Soybean Oil, and aggregates.
- In TSR tests, asphalt mixtures showed significant tensile strength improvements with innovative additives. The control mixture had a TSR of 82.9%, while LDPE (5%) yielded 83.4%. The most substantial improvement came from LDPE (5%) with 3% Oil, achieving an impressive TSR of 85.7%.
- The Dynamic Modulus test results reveal enhanced rutting resistance in asphalt mixtures with the incorporation of LDPE and a 3% oil additive. The Control mixture showed a Dynamic Modulus of 37 mPa at the lowest frequency, while LDPE (5%) + Control demonstrated a notable increase to 72 mPa. The LDPE (5%) with 3% Oil-modified mixture exhibited a remarkable improvement, reaching 214 mPa, highlighting the synergistic effects of LDPE and the oil additive. LDPE contributes to improved elastic and mechanical properties, while the oil additive acts as a rejuvenator.
- The SCB test reveals significant improvements in fracture characteristics and energy absorption by incorporating 5% LDPE and 3% oil into asphalt mixtures. The LDPE-modified mixture showed a stiffer break but a higher fracture point (6.2 mPa), while the LDPE (5%) with 3% Oil-modified mixture achieved a softer break, lower fracture point (5.4 mPa), and the highest fracture energy (518 J/m^2^).
- The HWT test assesses the resistance of asphalt mixtures to rutting and deformation under repetitive loading, simulating real-world traffic conditions. The LDPE (5%) + 3% Oil + Control mixture showed enhanced moisture resistance by exhibiting no sign of a stripping point throughout the test, indicating improved durability. In terms of rutting resistance, the LDPE (5%) + 3% Oil + Control mixture outperformed both counterparts at 20,000 cycles, with a deformation of 9.2 mm, representing a significant percentage reduction of approximately 39.7%.
- The Cantabro test results provide valuable insights into the aggregate shatter resistance of asphalt mixtures, emphasizing their cohesion and wear resistance. The LDPE (5%) + 3% Oil + Control mixture demonstrated the most significant enhancement with the lowest weight loss rate at 9.820%, showcasing the synergistic effects of LDPE and the introduced oil additive.
- The field test results offer crucial insights into the real-world performance of asphalt mixtures, specifically in terms of stress and deformation parameters. Analyzing the highest deflection in the surface layer, the LDPE (5%) + 3% Oil + Control mixture displayed superior stability with a 61% reduction in deflection compared to the Control mixture and a 19% improvement over the LDPE (5%) + Control mixture. Considering the lowest E1 (surface modulus), a key parameter indicating surface resistance to stress, the LDPE (5%) + 3% Oil + Control mixture demonstrated the highest E1 value at 1827 mPa, representing a notable 256% improvement over the Control mixture. Simultaneously, the LDPE (5%) + 3% Oil + Control mixture exhibited a low E2 stress of 1023 mPa, indicating a safe and well-distributed stress profile.
- While this study has advanced asphalt mixture performance with LDPE and an oil additive, it comes with limitations. Variations in climate and traffic conditions could affect outcomes, and long-term performance requires further observation. Future studies should explore diverse additives, consider varied environmental conditions, and assess economic feasibility. Additionally, examining the environmental impact and recyclability of modified mixtures would contribute to a comprehensive understanding of sustainability. This research serves as a foundation for ongoing exploration, aiming for advancements in creating durable, sustainable, and high-performance road pavement materials.

## Figures and Tables

**Table 1:** General properties of the recycled LDPE used in the research.

Property	Value
Polymer Type	LDPE
Source of LDPE	Recycled post-consumer plastic waste
Molecular Weight	90,000 g/mol
Melting Point	110 °C
Glass Transition Temperature	−80 °C
Density	0.92 g/cm^3^
Melt Flow Index	2.5 g/10 min
Chemical Structure	Linear hydrocarbon polymer
Particle Size Distribution	100–500 microns
Compatibility with Asphalt	Excellent
Environmental Impact	Sustainable, recycled material

**Table 3:** Summary of the Cantabro test.

	Specimen Weight (g)	Weight after the Test (g)	Loss Rate (within 20% Based on Drainage)
Controlled mix	1217.268	1037.646	15.051
LDPE (5%) + Control Mixture	1214.82	1066.716	12.434
LDPE (5%) + 3% Oil + Control Mixture	1216.248	1099.152	9.820

## Data Availability

Data are contained within the article.
